# Atrophy of the putamen at time of clinical motor onset in Huntington’s disease: a 6-year follow-up study

**DOI:** 10.1186/s40734-018-0069-3

**Published:** 2018-03-23

**Authors:** Emma M. Coppen, Jeroen van der Grond, Raymund A. C. Roos

**Affiliations:** 1 Department of Neurology (J3-R-162), Leiden University Medical Center, PO Box 9600, 2300 RC Leiden, The Netherlands; 2 Department of Radiology, Leiden University Medical Center, Leiden, The Netherlands

**Keywords:** Huntington’s disease, Structural MRI, Converters, Motor onset, Putamen, Longitudinal study

## Abstract

**Background:**

Striatal atrophy is detectable many years before the predicted onset of motor symptoms in premanifest Huntington’s disease (HD). However, the extent of these neurodegenerative changes at the actual time of conversion from premanifest to a motor manifest disease stage is not known. With this study, we aimed to assess differences in degree and rate of atrophy between converters, i.e. premanifest individuals who develop clinically manifest HD over the course of the study, and non-converters.

**Methods:**

Structural T1-weighted Magnetic Resonance Imaging (MRI) scans were used to measure volumes of seven subcortical structures. Images were acquired yearly over a maximum follow-up period of 6 years (mean 4.8 ± 1.8 years) in 57 participants (healthy controls *n* = 28, premanifest HD gene carriers *n* = 29). Of the premanifest HD gene carriers, 20 individuals clinically developed manifest HD over the course of the study, i.e. converters, whereas 9 individuals did not show any clinical signs. Differences between controls, converters and non-converters in volumetric decline over time were assessed using a one-way ANCOVA with age, gender and intracranial volume as covariates. All data were adjusted for multiple comparisons using Bonferonni correction.

**Results:**

The putamen showed a significant difference in volume at the time of conversion in the converters group compared to the non-converters group (adjusted *p* = 0.04). Although, volumes of all other subcortical structures were smaller at time of conversion compared to non-converters and controls, these differences were not statistically significant. Over time, rate of volumetric decline in all subcortical structures in converters did not significantly differ from non-converters.

**Conclusions:**

Putamen volume is smaller at the time of manifestation of motor symptoms compared with premanifest HD that not showed any clinical disease progression during the course of this 6-year follow-up study.

## Background

Huntington’s disease (HD), an autosomal-dominant inherited neurodegenerative disease, causes widespread atrophy throughout the cerebral cortex and the striatum [1, 2]. The disease is clinically characterized by a manifest stage in which motor disturbances, cognitive decline and psychiatric symptoms progress gradually [3].

After the detection of the cytosine-adenine-guanine (CAG) repeat expansion causing a mutation of the Huntingtin gene on chromosome four [4], special interest emerged to identify and investigate premanifest HD gene carriers; individuals with a CAG repeat expansion without motor symptoms, but who gradually will develop manifest HD.

It is currently well known that atrophy of the striatum is the hallmark sign of HD, and is already detectable in the premanifest disease stage, many years before the onset of motor symptoms [5–8]. In addition to the striatum, other subcortical grey matter structures and cortical brain areas also show early signs of atrophy in premanifest HD gene carriers, but are less pronounced [7, 9, 10].

In this respect, subcortical volumetric measures have shown to be clinically useful in the prediction of time to onset at which individuals convert from premanifest HD to motor manifest HD [5, 6, 11]. Furthermore, it has been suggested that in addition to volume differences between various disease stages, the rate of decline in striatal volume may be an important factor in disease progression [6, 7].

Although cortical and subcortical atrophy are considered early markers of the disease, it is not known whether the absolute reduction in striatal volume is indicative for clinical conversion from a premanifest disease stage without motor symptoms into clinically manifest HD. Moreover, it is not unlikely that the rate of decline in volume, rather that atrophy itself, is involved in the process of initiating conversion. Such data, obtained at the actual time of conversion, rather than comparing premanifest HD gene carriers with manifest HD gene carriers, may elucidate the underlying process that could initiate clinical motor conversion. Currently, subcortical volume changes that are present at the time of conversion into motor manifest HD have not yet been fully investigated.

The aim of the present study was to characterize differences in striatal and extrastriatal grey matter atrophy at the time of conversion, between premanifest HD gene carriers that progress into the manifest stage of the disease and premanifest individuals that do not show any clinical signs. Furthermore, we assessed the rate of atrophy by examining the degree of volume loss over time. Therefore, we have investigated premanifest HD gene carriers on a yearly basis that were followed over a period of 6 years. Providing insight into the brain changes that occur as premanifest HD gene carriers become clinically affected by the disease might guide the timing of future therapeutic intervention.

## Methods

### Participants

A total of 57 participants (28 healthy controls and 29 premanifest HD gene carriers) were included in this longitudinal retrospective cohort study. Participants included in our study had at least one follow-up visit and were seen annually from 2008 till 2014, with a maximum of seven visits.

Premanifest HD gene carriers included in our study had a genetically confirmed expanded CAG repeat of 40 or more and a disease burden score of more than 250, based on CAG length and age, to ensure a premanifest HD group close to disease onset [12]. The estimated predicted years to disease onset were calculated using a survival analysis formula based on the participants’ age at baseline and CAG repeat length [13]. At baseline, all premanifest HD gene carriers showed no clinical motor symptoms indicating manifest HD. This was defined as a total motor score (TMS) of 5 or less on the Unified Huntington’s Disease Rating Scale (UHDRS). The UHDRS-TMS is widely used for assessment of motor disturbances, ranging from 0 to 124, with higher scores indicating more increased motor impairment [14]. Certified movement disorder experts administered this scale and also assigned a score from 0 to 4 on the UHDRS Diagnostic Confidence Level (DCL), indicating the rater’s level of confidence that motor abnormalities reflect the presence of HD. The HD motor diagnosis is defined as a score of 4 on the DCL, meaning that the rater has ≥99% confidence that the participant shows motor abnormalities that are unequivocal signs of HD [7, 14]. In our study, 20 premanifest participants received a motor diagnosis with a rating of 4 on the UHDRS-DCL sometime during the course of the study, further referred to as ‘converters’. Partners and gene-negative relatives were recruited as healthy controls.

The local Medical Ethical Committee approved this study and written informed consent was obtained from all participants.

### MRI acquisition

From 2008 to 2014, all participants underwent structural magnetic resonance imaging (MRI) scanning each year with a maximum of 7 time points. Imaging was performed on a 3 Tesla MRI scanner (Philips Achieva, Best, the Netherlands) using a standard 8-channel whole-head coil. Three-dimensional T1-weighted images were acquired with the following parameters: TR = 7.7 ms, TE = 3.5 ms, flip angle = 8 °, FOV 24 cm, matrix size 224 × 224 cm and 164 sagittal slices to cover the entire brain with a slice thickness of 1.0 mm with no gap between slices, resulting in a voxel size of 1,07 mm × 1,07 mm × 1,0 mm.

### MRI post processing

All T1-weighted images were analyzed using software provided by FMRIB’s software library (FSL, version 5.0.8, Oxford, United Kingdom) [15].

Volumes of subcortical structures were measured for each time point using FMRIB’s Integrated Registration and Segmentation Tool (FIRST) [16]. Non-brain tissue was removed for all images using a semi-automated brain extraction tool implemented in FSL [17]. Subcortical regions include the accumbens, amygdala, caudate nucleus, hippocampus, pallidum, putamen and thalamus. T1-weighted images were registered to the MNI (Montreal Neurological Institute) 152-standard space image, using linear registration with 12 degrees of freedom [18]. Subsequently, segmentation of the subcortical regions was carried out using mesh models that are constructed from a large library of manually segmented images. Finally, a boundary correction was applied to prevent overlap with adjacent structures. Then, absolute volumes per structure were calculated. Visual inspection was performed during the registration and segmentation steps on randomly chosen images.

Whole brain intracranial volume, normalized for individual head size, was estimated with SIENAX [19]. Brain and skull images were extracted from the single whole-head input data. The brain images were then affine-registered to a MNI 152-space standard image [18], using the skull image to determine the registration scaling. Next, tissue-type segmentation with partial volume estimation was carried out in order to calculate the total volume of normalized brain tissue. Visual inspection of motion artifacts, registration and segmentation was performed for each brain-extracted image.

### Statistics

Statistical analyses were performed using the Statistical Package for Social Sciences (SPSS for Mac, version 23, SPSS Inc.). Demographic group differences at baseline were analyzed using the χ2 test for gender and independent-samples t-test for age, CAG repeat length, disease burden score, and UHDRS-TMS. Group differences in absolute subcortical volumes were analyzed using a one-way analysis of covariance (ANCOVA) with age, gender and normalized intracranial volume (ICV) as covariates.

To assess the individual change over time, a linear regression analysis was performed for each subcortical structure in each participant to calculate the linear regression slope. To account for an individuals’ total brain volume, we calculated for each individual and subcortical structure the ratio between subcortical volume and ICV by dividing the volume of the subcortical structure with the total ICV at each visit. With this volume/ICV ratio, we constructed a linear fitted coefficient that indicates the estimated change (increase or decrease) in volume per participant for every additional year adjusted for total brain volume. Then, this regression coefficient was used as a dependent variable in a one-way ANCOVA with age and gender as covariates.

The significance level was set at *p* < 0.05. Bonferonni correction for post-hoc analyses was performed to correct for multiple comparisons.

## Results

### Demographic characteristics

Demographic and clinical group characteristics at baseline are shown in Table 1. The mean follow-up period was 4.8 years (SD 1.8 years, range 0.9–6.6 years). Longitudinal data was collected for two or more years in 55 of the 57 participants. For the two remaining participants, the follow-up period was 1 year. Of all participants, 36 (63%) completed a follow-up period of 6 years.

**Table 1:** Demographic and clinical baseline characteristics

	Premanifest HD			Controls
	Non-converters	Converters	Combined	
Number of participants	9	20	29	28
Gender (male/female)	3/6	9/11	12/17	13/15
Age (years)	41.6 (6.5)	44.2 (8.7)	43.3 (8.0)	48.6 (7.9) ^†^
CAG repeat length	42.4 (1.8)	43.8 (2.6)	43.4 (2.4)	–
Disease burden score	286 (56.9)	352 (61.3)^*^	332 (66.6)	–
UHDRS - TMS	1.9 (1.8)	2.7 (1.3)	2.4 (1.5)	2.6 (2.4)

Data is given in mean (standard deviation). Premanifest HD gene carriers were divided into non-converters with a Diagnostic Confidence Level (DCL) below 4 and converters who progressed to manifest HD rated as a DCL of 4. CAG: cytosine-adenine-guanine. UHDRS-TMS: Unified Huntington’s Disease Rating Scale – Total Motor Score. Disease burden score = age x (CAG length − 35.5) by Penney et al. [12]

^*^Significantly different between converters and non-converters at *p* < 0.05

^†^Significantly different between controls and premanifest HD combined group at *p* < 0.05

There were no significant differences in gender and total motor score at baseline between controls and the whole group of premanifest HD gene carriers. Controls were significantly older compared to the premanifest HD group (t(55) = 2.48, *p* = 0.016).

The premanifest HD group was subsequently divided into converters (*n* = 20 with DCL = 4) and non-converters (*n* = 9 with DCL < 4). After baseline, converters had a median time of progression into manifest HD of 4.0 years (SD 1.5 years). Compared to non-converters, converters had a significantly higher mean disease burden score (t(27) = − 2.73, *p* = 0.011) at baseline.

### Subcortical volume at time of conversion

**Fig. 1:**
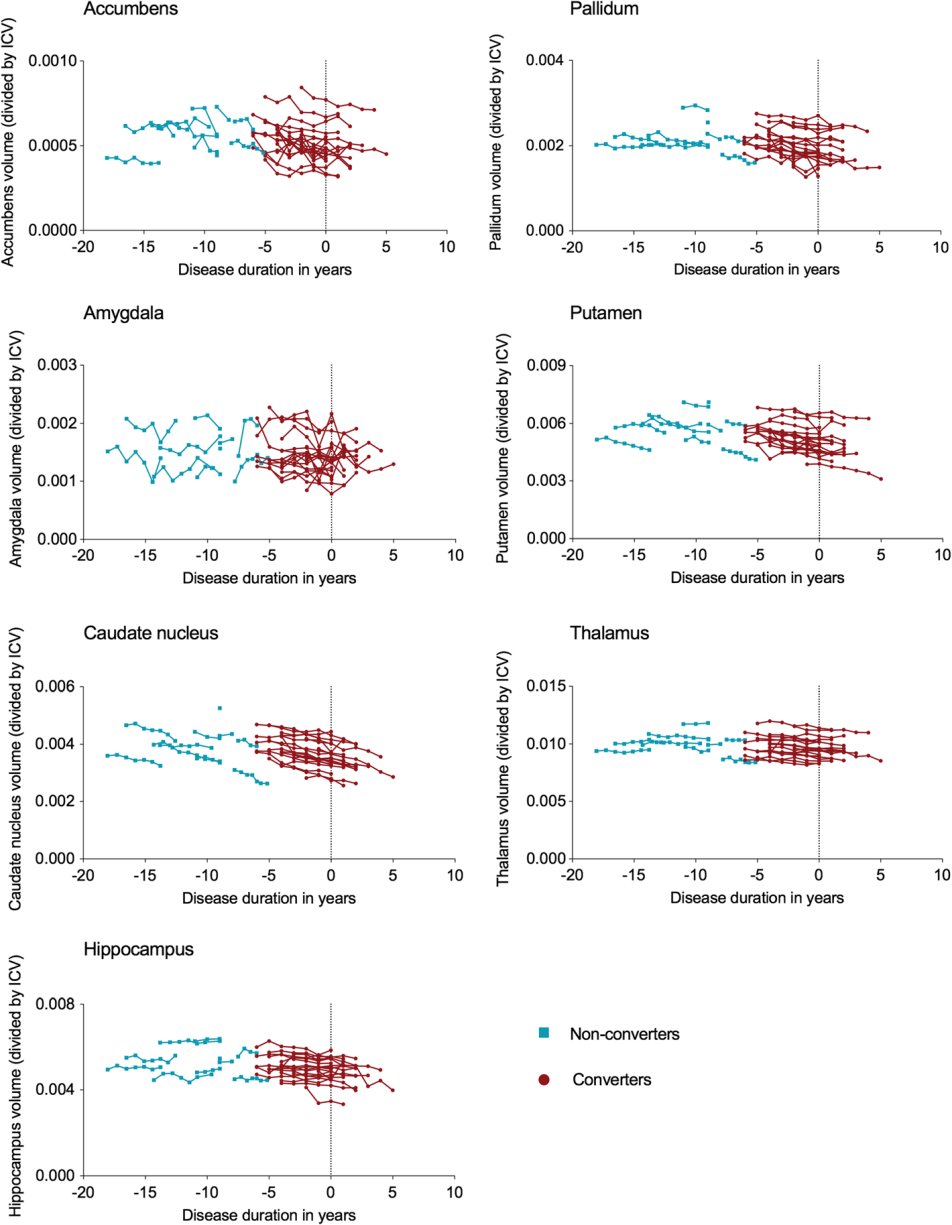
Subcortical volume change over time. Individual volumes over time in premanifest HD for all seven subcortical structures. Disease duration (years) was calculated for converters with time of conversion based on the Diagnostic Confidence Level (DCL) of 4. For non-converters, estimated time to disease onset was calculated using the survival analysis formula of Langbehn et al. [13]. ICV: Intra-Cranial Volume

Mean volumes of seven subcortical structures (accumbens nucleus, amygdala, caudate nucleus, hippocampus, pallidum, putamen and thalamus) were calculated for converters at the time of conversion (Table 2), whereas absolute volume changes are shown in Fig. 1.

**Table 2:** Subcortical volumes

	Controls	Premanifest HD gene carriers		*p* – value non-converters vs. converters^a^
		Non-converters	Converters	
Accumbens	0.92 (0.21)	0.86 (0.15)	0.72 (0.18) ^*^	0.566
Pallidum	3.39 (0.44)	3.15 (0.50)	2.78 (0.54) ^*^	0.149
Amygdala	2.19 (0.36)	2.31 (0.49)	2.01 (0.51)	0.157
Putamen	9.31 (1.30)	8.32 (1.34)^*^	7.24 (1.05) ^*^	*0.040*
Caudate nucleus	6.67 (0.88)	5.65 (0.78)^*^	5.15 (0.73) ^*^	0.367
Thalamus	14.79 (1.32)	14.76 (1.40)	13.87 (1.51)	0.402
Hippocampus	7.75 (0.80)	7.85 (0.97)	7.15 (0.86)	0.178

Uncorrected mean (standard deviation) subcortical volumes in ml are displayed. For converters, volumes at time of conversion are measured. For non-converters and controls, mean volumes across visits were calculated. Results of a one-way analysis of covariance (ANCOVA) for group differences with age, gender and intracranial volume as covariates. Significant difference between converters and non-converters is displayed in *Italic*.

^a^Post-hoc analyses were adjusted for multiple comparisons using a Bonferonni correction

^*^Significant difference compared to controls, *p* < 0.05

Converters showed a lower mean volume at time of conversion for all subcortical structures compared with the mean volume across visits in controls and non-converters. After correction for age, gender and intracranial volume, and adjustment for multiple comparisons, the accumbens nucleus (F(2,51) = 4.02, *p* = 0.020, η_p_^2^ = 0.14), pallidum (F(2,51) = 5.46, *p* = 0.007, η_p_^2^ = 0.18), putamen F(2,51) = 15.96, *p* < 0.001, η_p_^2^ = 0.39), and caudate nucleus (F(2,51) = 16.84, *p* < 0.001, η_p_^2^ = 0.40) were all smaller in converters compared with controls.

Volumes of the caudate nucleus and putamen in non-converters were also smaller compared to controls (*p* = 0.020 and *p* = 0.044 respectively). Converters only had a significantly smaller putamen volume at time of conversion compared with non-converters (*p* = 0.040).

### Subcortical volume change over time

The caudate nucleus (F(2,50) = 4.37, *p* = 0.018, η_p_^2^ = 0.15) demonstrated a significantly steeper decrease in volume in both converters and non-converters compared with controls (Table 3, Fig. 1). The pallidum showed a higher decline in volume over time in converters compared to controls (F(2,50) = 4.61, *p* = 0.015, η_p_^2^ = 0.16). No significant differences in atrophy rate for any subcortical structure were found between converters and non-converters.

**Table 3:** Mean subcortical volume change in ml per year

	Controls	Premanifest HD gene carriers	
		Non-converters	Converters
Accumbens	- 0.018 (0.027)	- 0.035 (0.046)	- 0.020 (0.014)
Pallidum	0.006 (0.041)	- 0.054 (0.056)	- 0.049 (0.043)^*^
Amygdala	- 0.0012 (0.107)	0.031 (0.109)	- 0.020 (0.063)
Putamen	- 0.022 (0.082)	- 0.165 (0.128)	- 0.123 (0.066)
Caudate nucleus	- 0.023 (0.048)	- 0.086 (0.079)^*^	- 0.125 (0.052)^*^
Thalamus	- 0.067 (0.099)	- 0.015 (0.090)	- 0.106 (0.070)
Hippocampus	- 0.010 (0.123)	0.029 (0.041)	- 0.066 (0.084)

Mean individual linear regression coefficients (standard deviation) indicate the change (increase or decrease) in volume for each subcortical structure for every additional year. In the statistical regression analyses, the ratio between subcortical volume and total intracranial volume at each measurement was used to account for an individuals’ brain volume. A one-way analysis of covariance (ANCOVA) for group differences was performed with age and gender as covariates. Post-hoc analyses were adjusted for multiple comparisons using a Bonferonni correction

^*^Significantly different compared to controls, *p* < 0.05

## Discussion

Our results showed that putamen volume is reduced in individuals that converted to the manifest disease stage compared to individuals that did not show any clinical disease progression during the study period of 6 years. Although atrophy rate over time of the pallidum and caudate nucleus was higher in converters compared with controls, no differences in atrophy rate were found between converters and non-converters for any of the subcortical structures.

The putamen is essential for regulation of movements and for learning and performance of motor skills. As the clinical diagnosis of HD is based on the presence of unequivocal motor signs, our results suggest that the putamen undergoes degeneration, when premanifest individuals approach clinical motor onset.

To our knowledge, one other study specifically focused on longitudinal brain changes in premanifest HD that converted into a manifest disease stage [11]. Their results showed that putamen volume could be used to improve the prediction of disease onset in addition to CAG repeat length and age [11]. We provide evidence of atrophy of the putamen at the actual time of clinical motor onset, instead of using predicted data. Nevertheless, our findings that premanifest individuals have a smaller volume of the putamen at the time of clinical motor onset confirm this suggestion of using putamen volume as a predictor for disease onset. The large multi-center observational Track-HD study also focused on identifying predictors of disease progression in early HD and premanifest HD gene carriers [7]. Here, baseline and longitudinal caudate nucleus, putamen and grey matter volumes showed a strong predictive value for the risk of future clinical diagnosis in a premanifest individual [7]. In our study, we found no difference in the rate of subcortical volume loss over time between converters and non-converters. An explanation for this finding could be that other factors, such as environmental, biochemical and genetic aspects, might play a more substantial role in clinical motor onset than striatal volume loss. Another explanation might be that the number of non-converters in our study was too minimal to detect such specific differences.

Compared with controls, the whole premanifest HD group did show a more rapid decline in volume loss over time for the pallidum and caudate nucleus. This finding is consistent with previous longitudinal studies [10, 20]. However, other longitudinal studies also showed that in premanifest HD gene carriers, the volume of the caudate nucleus declines more rapidly over time than the putamen volume [6, 21], which is contradictory to our findings. Specifically, the results of our study suggest that the *degree* of decline in subcortical volume might not be a reliable marker for clinical motor onset in premanifest HD, as we did not find any differences in rate of decline between converters and non-converters over time.

Our findings are strengthened by the fact that we used data of the actual time of clinical motor onset in the converter group rather than predicted data. Also, many studies that have been performed to date are cross-sectional and compare brain atrophy between different disease stages, such as premanifest and early manifest HD, to measure disease progression.

Striatal atrophy is one of the most recognized neurodegenerative signs in HD, and is associated with chorea severity. [22, 23] In our study, we have therefore focused on the progression of subcortical changes in premanifest HD. In manifest HD gene carriers, oculomotor dysfunction, however, was related to volume changes in the occipital cortex. [23] It could therefore be of great interest to examine longitudinal cortical changes in relation to clinical symptoms in larger clinical trials. To assess clinical disease progression in premanifest HD, we defined clinical motor onset as the time that certified raters had a confidence of ≥99% that the participant showed motor abnormalities that are unequivocal signs of HD (measured with a DCL score of 4). Participants were rated each year at their annual follow-up visit, which included the MRI scan. We acknowledge that this might be a conservative classification of clinical motor onset, as other studies also defined a decline in functional capacity or increase in total motor score as disease progression [20, 24]. Still, the DCL score showed to be stable over time and was used previously to monitor disease progression [7, 11, 25]. Therefore, it would be interesting to assess volumetric changes over time in premanifest non-converters with DCL scores between 1 and 3 (e.g. non specific motor abnormalities or motor signs that are likely signs of HD), but our cohort consisted of a relatively small number of non-converters. In addition, this small number of non-converters might explain the non-significant findings in our longitudinal analyses. Future studies with larger samples sizes are necessary to confirm and extend the findings of our study. Then, there is also a possibility to assess if specific brain regions, independent of certain classifications into groups, can predict progression of motor symptoms. In this way, large longitudinal studies with longer follow-up periods can provide a better understanding of disease progression in HD as this might guide the timing of future therapeutic intervention.

## Conclusion

In summary, we provide new insights in the difference in putamen volume between HD patients at the time of clinical motor onset and premanifest individuals that do not show any clinical progression. Our longitudinal study in premanifest HD demonstrates that the degree of atrophy in putamen volume, rather than rate of decline in volume, is involved in the process of conversion into clinically motor manifest HD.

This implies that putamen volume might be a suitable neuroimaging outcome measure for clinical trials as a marker for disease onset.

## Abbreviations

ANCOVA: Analysis of Covariance
CAG: Cytosine-adenine-guanine
DCL: Diagnostic Confidence Level
FIRST: FMRIB’s Integrated Registration and Segmentation Tool
FSL: FMRIB’s Software Library
HD: Huntington’s disease
ICV: Intracranial Volume
MNI: Montreal Neurological Institute
MRI: Magnetic Resonance Imaging
SPSS: Statistical Package for Social Sciences
TMS: Total Motor Score
UHDRS: Unified Huntington’s Disease Rating Scale
